# Fine Mapping of a Degenerated Abdominal Legs Mutant (*E^dl^*) in Silkworm, *Bombyx mori*

**DOI:** 10.1371/journal.pone.0169224

**Published:** 2017-01-12

**Authors:** Honglei Wang, Xiaoling Tong, Meijing Liu, Hai Hu, Zhiquan Li, Zhonghuai Xiang, Fangyin Dai, Cheng Lu

**Affiliations:** State Key Laboratory of Silkworm Genome Biology, Key Laboratory for Sericulture Functional Genomics and Biotechnology of Agricultural Ministry, Southwest University, Chongqing, China; Institute of Plant Physiology and Ecology Shanghai Institutes for Biological Sciences, CHINA

## Abstract

In insects, abdominal appendages, also called prolegs, vary due to adaptive evolution. Mutations on prolegs within species provide insights to better understand the mechanisms underlying appendage development and diversity. In silkworm *Bombyx mori*, extra-crescents and degenerated abdominal legs (*E*^*dl*^) mutant, belonging to the *E* pseudoallele group, is a spontaneous mutation that adds crescents and degenerates prolegs on the third abdominal segment (A3). This mutation may be a homeotic transformation of A3 to A2. In this study, the *E*^*dl*^ locus was mapped within approximately a 211 Kb region that is 10 Kb upstream of *Bmabdominal-A* (*Bmabd-A*). RT-quantitative PCR (RT-qPCR) and Western blot analysis of *Bmabd-A* expression showed a slight but significant decrease, while the expression of *BmUltrabithorax* (*BmUbx*) was up-regulated in the *E*^*dl*^ mutant compared to wildtype (Dazao). Moreover, we also found that BmDistal-less (BmDll), which regulated the development of distal proleg structures, was missing at the tips of the A3 prolegs in the *E*^*dl*^ mutant compared to BmDll expression in normally developed prolegs in both the wildtype and mutant. Collectively, we identified approximately a 211 Kb region in the *E*^*dl*^ locus that regulates *BmUbx* and *Bmabd-A* expression and found that changes in *BmUbx* and *Bmabd-A* expression may lead to the loss of distal proleg structures in *B*. *mori*.

## Introduction

Morphological characteristics of insects are surprisingly variable. Particularly, the appendage number, morphology and position vary largely within and between species. Studies on the mechanisms underlying appendage development and diversity provide insights into the evolutionary process of arthropods [1–3].

Each thoracic segment, on the ventral side of an insects’ trunk, contains appendages called thoracic legs. The appendages on the abdominal segments are known as prolegs and their numbers vary markedly [4]. Segmental identities of these legs are determined by several Homeotic genes [5], which consist of a tandem gene cluster where the order of genes is co-linear with their expression pattern on the body axis [6]. The development of prolegs is mainly regulated by *Ultrabithorax* (*Ubx*) and *abdominal-A* (*abd-A*). For example, in the dipteran, *Drosophila melanogaster*, *abdominal-B* (*abd-B*), *abd-A* and *Ubx* repress proleg development [7–9]; in the flour beetle, *Tribolium castaneum*, *Ubx* does not repress but modifies the morphology of prolegs on abdomen 1 (A1), *abd-A* represses the development of proleg on the posterior abdominal segments [10]; in the lepidopteran, *Manduca sexta*, *Ubx* represses the development of prolegs on the anterior abdomen, but *abd-A* does not repress the development of prolegs because robust expression of *abd-A* is found in proleg primordia of A3-A6, which develop into prolegs [11]. Finally, in the lepidopteran, *Bombyx mori*, *BmUbx* and *Bmabd-B* repress the development of prolegs on the anterior and posterior abdominal segments, respectively; *Bmabd-A*, on the other hand, is known to play an important role in the development of prolegs on the intermediate abdominal segments, and RNAi of *Bmabd-A* results in the deletion of prolegs [12–17].

Studies in *Drosophila* have shown that the bithorax complex (BX-C) contains many *cis*-regulatory elements and some non-protein coding transcripts, which work together to control neighboring Hox gene expression [18–20]. The *cis*-regulatory elements are organized into several regulatory domains, which determine specific gene expression in different abdominal segments, and mutations of these domains can lead to homeotic transformation [21, 22]. In silkworm, the corresponding region extends to 620Kb (International Silkworm Genome Consortium, 2008) and contains only three protein coding genes, which are likely responsible for more than 30 mutant phenotypes [16, 17, 23]. However, only two mutants, *E*^*Cs*^*-l* and *E*^*kp*^*-*1, have been mapped to the region [15, 23], which is referred to as the *E* locus. Additional studies associated with the more than 30 mutants are needed to characterize the corresponding functional units.

In silkworm, more than 30 mutant phenotypes exist in the *E* locus, which is analogous to BX-C in *D*. *melanogaster* [17, 24]. The phenotypic characteristics of these mutants are associated with legs, markings, segmentation, genitalia as well as the nervous system [25].The extra crescents and degenerated abdominal legs (*E*^*dl*^) mutant has degenerated prolegs without distal proleg structures and shows a homeotic transformation of A3 to A2; thus, it may be a good model to study Hox function in distal proleg development. In this study, the *E*^*dl*^ locus was narrowed down to approximately a 211 Kb region that is 10 Kb upstream of *Bmabd-A*. Expression analysis revealed that *BmUbx* was up-regulated and *Bmabd-A* was down-regulated, suggesting a homeotic transformation of A3 to A2 in the *E*^*dl*^ mutant. The expression of Dll was also changed, providing evidence for the function of Hox genes in distal proleg development.

## Materials and Methods

### Silkworm strains

*E*^*dl*^ and Dazao strains were obtained from the Silkworm Gene Bank in Southwest University, China. The larvae were reared on fresh mulberry leaves under a 12 h light/12 h dark photoperiod at 25°C. Eggs of the two strains were hatched at 25°C and maintained in conditions with adequate humidity and embryos were staged according to Takami [26].

### DNA extraction

Genomic DNA was obtained from the whole body of fourth instar larvae. Samples were first powdered in liquid nitrogen and then digested in DNA extraction buffer (pH 8.0, 10 mM Tris-HCl, 0.1 M ethylenediaminetetraacetic acid (EDTA), 0.5% sodium dodecyl sulphate (SDS)) with 100 μg/mL proteinase K. After digestion for 5–8 h at 50°C, phenol: chloroform extraction was performed and the resulting DNA was suspended in TE buffer (elution buffer, pH 8.0, 10 mM Tris-HCl, 1 mM EDTA).

### Mapping of the *E*^*dl*^ locus

Dazao (+/+) and *E*^*dl*^ (*E*^*dl*^/*E*^*dl*^) were used for the fine mapping. F_1_ offspring were produced by a single-pair cross between a female (*E*^*dl*^) and a male (Dazao). For linkage analysis, 20 BC_1_F progenies (10 wildtype and 10 mutant) from the cross (*E*^*dl*^ × Dazao)F_1_♀ × *E*^*dl*^♂ were used; for recombination analysis, 1205 BC_1_M progenies from the cross *E*^*dl*^♀ × (*E*^*dl*^ × Dazao)F1♂ were used. Based on the mapping of *E*^*Cs*^*-l* and *E*^*kp*^*-*1 mutants in the *E* pseudoallele group [15, 23], new primers were designed to narrow down the *E*^*dl*^ locus, and markers with polymorphism were used for genotyping the 1205 BC_1_M progenies. This analysis requires the silkworm 9 × assembly genome database (http://www.silkdb.org/silkdb/, SilkDB), BLAST [27] and primer 5. The primer sequences are shown in Table 1.

**Table 1:** Sequences of primers used in this study.

Object	Name of the markers	Forward primer(5′−3′)	Reverse primer(5′−3′)
Linkage analysis	D1	CGCCAATTAGGCTTCCATCTA	TTCACTCGCTTTCGCTTGTT
	D2	TGCCGCTTATTAGGTCAGATTGT	TCCCTGAATGATTCCCGATG
	D3	CTTACGCAAAACCGCACC	CCAAACACGAATCGCCTAA
	D4	GAAAATTCTCACACAAAGACGCC	AACAAACGGTAAAGCACTCGCC
	D5	CAGATTCTCCCGTGTTTTCG	CCTTACATCCACAGTATCATTCGT
	D6	GGATAATAGTGGCTGGTGTA	AGTGAAGCGATTGTGGTAAG
RT-qPCR	*Bmabd-A*	CGTGGTCTGCGGTGAGTTC	GGCGTGTGCTATTTCTATCCTG
	*BmUbx*	CGTGGTCTGCGGTGAGTTC	GGCGTGTGCTATTTCTATCCTG
	sw22934	TTCGTACTGCTCTTCTCGT	CAAAGTTGATAGCAATTCCCT
	*RPL3*	CGGTGTTGTTGGATACATTGAG	GCTCATCCTGCCATTTCTTACT

### Reverse transcription–quantitative PCR (RT-qPCR)

Embryos of Dazao and *E*^*dl*^ were dissected at stage 20. Total RNA from embryos was extracted using a MicroElute Total RNA Kit (Omega Bio-Tek, Norcross, GA, USA) and cDNA was obtained using the PrimeScript RT Reagent Kit with gDNA Eraser (TaKaRa, Dalian, China). An ABI Prism 7000 Sequence Detection System (Applied Biosystems, Foster City, CA, USA) with the SYBR Premix ExTaq Kit (TaKaRa) was employed to detect mRNA levels of *Bmabd-A* and *BmUbx* in Dazao and *E*^*dl*^ mutant. All operations with instruments and kits were performed according to the manufacturer’s instructions. The eukaryotic translation initiation factor 4A (BmMDB probe ID: sw22934) and ribosomal protein L3 (*RPL3*) were used as internal controls. The melt curves, melt peaks and standard curves of the *BmUbx* and *Bmabd-A* primers are shown in S1 Fig. Each sample had three biological replicates and each experiment had three technical replicates. The results are presented as mean ± SD. Statistical analysis was carried out through Student’s *t*-test. The primers used in RT-qPCR are listed in Table 1.

### Protein isolation and western blotting

Embryos at stage 20 were used. Protein isolation, quantification and Western blot analysis were as reported by Chen [15]. Antibodies used in Western blot analysis were provided by Chen [15].

### Immunocytochemistry

For immunostaining, *B*. *mori* embryos at stage 20 were dissected and fixed in 4% paraformaldehyde for 30 min at 4°C. After fixation, the samples were washed in wash buffer (pH 6.8, 50 mM Tris, 150 mM NaCl, 0.5% (octylphenoxy) polyethoxyethanol (IGEPALCA-630), 1 mg/ml bovine serum albumin) and then incubated overnight at 4°C in a 1:200 dilution of anti-Dll [28]. The specimens were then incubated in a 1:1000 dilution of secondary antibody (Alexa Fluor 488, Beyotime) for 3h at 4°C. The stained embryos were then mounted in medium (Sigma, St. Louis, MO, USA) and were observed through a fluorescence microscope (BX51TRF, Olympus, Tokyo, Japan).

## Results

### Phenotypic characteristics of the *E*^*dl*^ mutant

**Fig 1:**
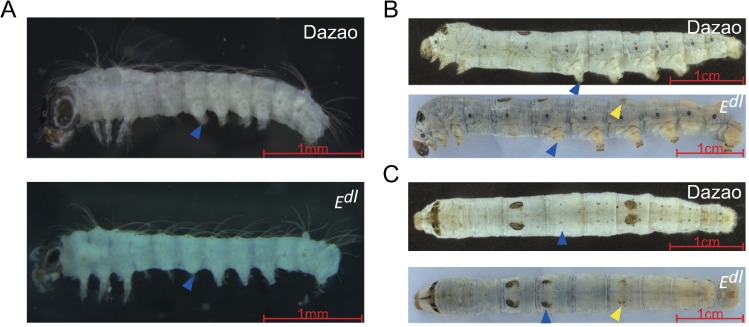
Phenotype of wildtype (Dazao) and extra-crescents and degenerated abdominal legs (*E*^*dl*^) mutant. Lateral views of embryos (A), and dorsal and lateral views of larvae (B, C) are shown with head on the left side. (A, B) Prolegs on the third abdominal segment (A3) are degenerated in *E*^*dl*^ compared to those in Dazao. Blue arrowheads indicate prolegs on A3 in *E*^*dl*^ and Dazao. (C) Extra crescents are arising on the dorsal side of A3 in *E*^*dl*^. Blue arrowheads indicate the marking in the dorsal part of A3 in *E*^*dl*^ and Dazao. Yellow arrowheads indicate the marking in the dorsal part of A8 in *E*^*dl*^. Bars A 1 mm; B, C 1 cm.

Compared to wildtype Dazao, the phenotype of *E*^*dl*^ mutant has variations in the abdominal appendages and crescents. In embryo and larval stages, prolegs on the third abdominal (A3) segment are degenerated; specifically, the distal claws in the prolegs are missing (Fig 1A and 1B). A pair of crescents arises on the dorsal part of A3 during the larval stage (Fig 1C). *E*^*dl*^ is the only recessive mutant in the *E* pseudoallele group and was previously described as a dominant mutant. We also show the presence of two star spots on the A8 segment in our *E*^*dl*^ mutant (Fig 1B and 1C), while there are no star spots in the *E*^*Dl*^ mutant from Japan indicating differences in the genetic background of the two mutants (http://shigen.nig.ac.jp/silkwormbase/ViewGeneDetail.do?id=68).

### Fine mapping of the *E*^*dl*^ locus

**Fig 2:**
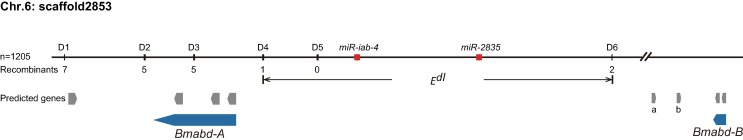
Mapping of the extra-crescents and degenerated abdominal legs (*E*^*dl*^) locus in linkage group 6. Gray arrows below the map are genes predicted in silkDB. The blue arrows represent verified genes. Symbols above the map are markers used for narrowing the *E*^*dl*^ locus. The locus was narrowed down between markers D4 and D6 in the intergenic region upstream of *Bmabdominal-A* (*Bmabd-A*), containing two microRNAs, miR-iab-4 and mir-2835. Predicted genes a and b indicate *BGIBMGA006488* and *BGIBMGA006489*, respectively. Red lines indicate the two microRNAs. Numerals below the map are recombinants in 1205 BC_1_M individuals.

To identify the candidate locus responsible for the *E*^*dl*^ mutant, we mapped the *E*^*dl*^ locus within approximately a 211 Kb region with newly designed markers, using 1205 BC_1_ individuals (a cross between *E*^*dl*^♀ × (*E*^*dl*^ × Dazao)F1♂). The *E*^*dl*^ locus was narrowed down between markers D4 and D6, and was tightly linked to marker D5 (Fig 2). As a result, no protein coding gene was predicted in this region (silkDB) but two ncRNAs, mir-iab-4 and mir-2835, were identified (Fig 2) [29–31]. However, the genomic sequences of pre-mir-iab-4 and pre-mir-2835 in *E*^*dl*^ were identical to Dazao (GenBank accession numbers: KX344456~KX344459). One side of the region was approximately 10 Kb upstream of the *Bmabd-A* gene while the other side of the region was more than 40 Kb upstream of two predicted genes, *BGIBMGA006488* and *BGIBMGA006489*, and approximately 101 Kb downstream of the *Bmabd-B* gene (Fig 2). However, the Hox gene cluster in insects is conserved, and no protein coding gene has been identified in the genomic region between *abd-A* and *abd-B* in *D*. *melanogaster* and the corresponding region in other insect orthologs. Moreover, ESTs and transcripts of the two predicted genes could not be detected in NCBI or SilkTransDB. Thus, *Bmabd-B* was the functional gene nearest to the other side of the region and we narrowed down the region between *Bmabd-A* and *Bmabd-B* (Fig 2). Considering the *E*^*dl*^ phenotype and neighboring Hox gene function, we examined the expression levels of *BmUbx* and *Bmabd-A* in the *E*^*dl*^ mutant.

### Expression profiles of *BmUbx* and *Bmabd-A*

**Fig 3:**
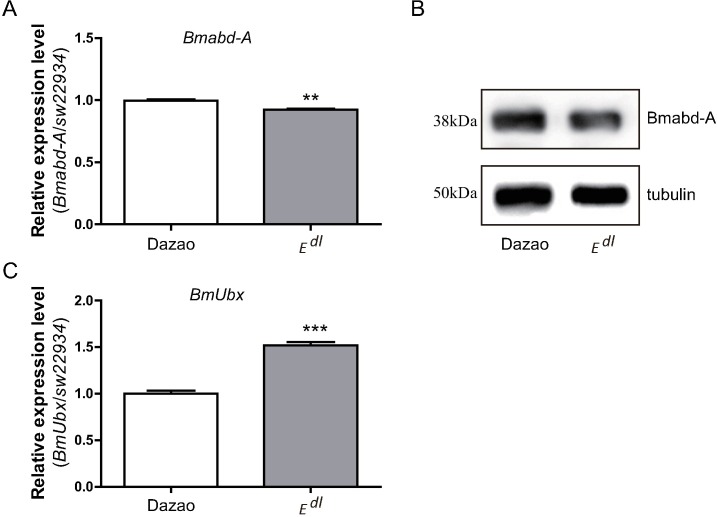
Expression of *BmUbx* and *Bmabd-A* in wildtype and *E*^*dl*^ mutant in stage 20 embryos. (A, C) RT-qPCR analysis of *Bmabd-A* and *BmUbx*. *Bmabd-A* was significantly down-regulated and *BmUbx* was up-regulated in *E*^*dl*^ mutants (Bars indicate mean values ± SD, Student’s *t*-test. *, P<0.05. n = 3). SW2934 was used as the internal control. (B) Western blot analysis of Bmabd-A. Expression level of Bmabd-A protein was decreased in *E*^*dl*^ mutants. Tubulin was used as the control to monitor equal loading of total proteins.

We determined the expression profiles of *BmUbx* and *Bmabd-A* in stage 20 embryos, which is a significant stage for proleg development. RT-qPCR and western blot analyses revealed a marginal decrease in the expression level of *Bmabd-A* in the *E*^*dl*^ mutant compared to wildtype (Dazao). In contrast, *BmUbx* was significantly up-regulated in the *E*^*dl*^ mutant (Fig 3 and S2 Fig). As reported in previous studies, blocking *Bmabd-A* through RNAi caused defects in proleg development. On the other hand, the ectopic expression of *Bmabd-A* resulted in the development of extra prolegs, while *BmUbx* repressed the development of proleg [12–16]. These results together indicate that the *E*^*dl*^ phenotype is a homeotic transformation of A3 to A2.

### Expression pattern of BmDistal-less (BmDll)

**Fig 4:**
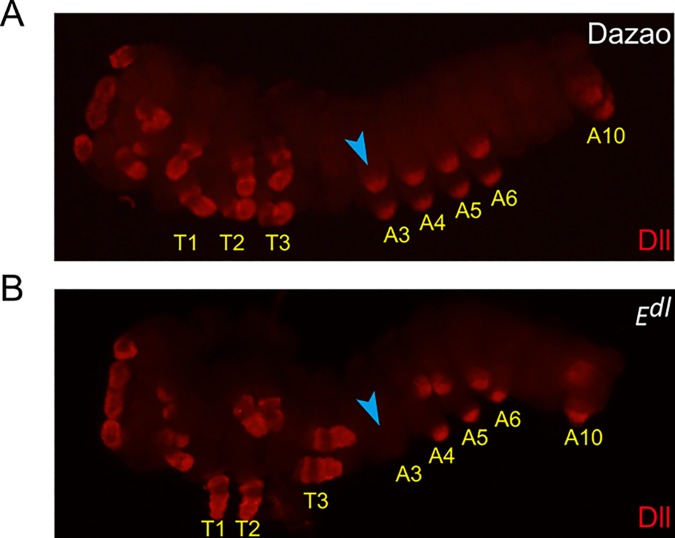
*Bombyx* Distal-less (BmDll) antibody staining of embryos at stage 20 in Dazao and *E*^*dl*^ mutant. BmDll staining (red) was detected in the distal part of all developed appendage primordia. T1, T2 and T3 represent three thoracic segments. The image was focused on abdominal appendages. Blue arrowheads were used for marking the prolegs on A3 segment. (A) BmDll staining was detected in the distal part of prolegs on A3-A6 and A10 segments in Dazao. (B) BmDll staining was not detected in the distal part of prolegs on A3 and was normally detected on A4-A6 and A10 segments in *E*^*dl*^. The prolegs on A3 were also degenerated compared to prolegs on A4-A6 in *E*^*dl*^.

The development of distal limb structures is controlled by the expression of *Dll* in arthropods [1, 32–34]. In addition, *BmDll* was shown to be present in the proleg primordial tips in silkworm [35]. Since the *E*^*dl*^ mutant possesses degenerated prolegs without distal limb structures on A3, we performed whole-mount BmDll antibody staining to detect whether its expression pattern was changed. In wildtype, BmDll was detected in the distal part of A3-A6 proleg primordia (Fig 4A). However, in the *E*^*dl*^ mutant, it was detected only in the distal part of A4-A6 and was not detectable in A3 (Fig 4B). These results indicate that the *BmDll* expression pattern is changed in the *E*^*dl*^ mutant and that *BmDll* is responsible for the development of distal limb structures.

## Discussion

In silkworm, the *E*^*dl*^ mutant belonging to the *E* pseudoallele group has crescents on the dorsal side and degenerated abdominal legs on the ventral side of A3, and it is the only recessive mutant in the *E* group. The degenerated abdominal legs seem to arise due to the incomplete transformation of A3 to A2, and the crescents seem to appear due to the complete transformation of A3 to A2. Therefore, it is likely that the *E*^*dl*^ phenotype is due to the homeotic transformation of A3 to A2. In this study, we narrowed down the *E*^*dl*^ locus to approximately a 211 Kb region between *Bmabd-A* and *Bmabd-B*. The neighboring *Bmabd-A* and *BmUbx* are regulated differentially that aligns well with their contrasting functions; *Bmabd-A* is down-regulated and is involved in proleg development while *BmUbx* is up-regulated and represses the development of prolegs [15, 17]. Collectively, these may explain the origin of the *E*^*dl*^ phenotype.

In silkworm, the *E* pseudoallele group contains more than 30 mutant phenotypes. In this study, the *E*^*dl*^ locus was delimited between *Bmabd-A* and *Bmabd-B*. Within this region in the *E*^*dl*^ locus, we found two miRNAs, mir-iab-4 and mir-2835, which were potential functional units. Sequence analysis of pre-mir-iab-4 and pre-mir-2835 in *E*^*dl*^ showed no differences compared to Dazao (GenBank accession numbers: KX344456~KX344459). The functions of these two miRNAs in silkworm need further investigation. Moreover, two other mutant phenotypes in the *E* pseudoallele group, *E*^*Cs*^*-l* and *E*^*kp*^*-*1, were also mapped to relatively small regions in the Hox gene cluster. The *E*^*kp*^-1 locus was restricted within a 220 kb region using 2396 individuals, containing the *Bmiab-4* locus and *Bmabd-A* gene, and the *E*^*Cs*^*-l* locus was mapped within approximately a 68 kb region upstream of *Bmabd-A*, including the *Bmiab-4* locus, while the sequence analysis of the transcripts in the narrowed regions showed synonymous mutations in the ORF of the *Bmabd-A* gene in *E*^*kp*^*-*1 and no difference at the *Bmiab-4* locus in *E*^*Cs*^*-l* and *E*^*kp*^*-*1 compared to wildtype [15, 23]. In *D*. *melanogaster*, the BX-C region contains many potential *cis*-regulatory domains and ncRNAs, and these functional units control the precise spatiotemporal expression patterns of BX-C genes [18–20]. As the *E* locus in silkworm is analogous to the BX-C region in *D*. *melanogaster*, and the *E*^*kp*^-1, *E*^*Cs*^*-l* and *E*^*dl*^ locus was narrowed to relatively small regions with no functional elements responsible for the mutant phenotypes identified, we expect that these regions could contain diverse potential functional elements such as *cis*-regulatory elements and ncRNAs. Further studies should be performed to identify and characterize new functional elements in the regions. Characterization of the *E*^*dl*^ mutant in this study and two other mutants may provide the foundation for the finding of functional elements.

Previous studies have shown that *abd-A* specifies the abdominal appendages in lower insect orders but represses the development of abdominal appendages in middle insect orders, highly developed dipterans, and *Precis coenia*, a lepidopteran [10, 36–38]. The presence of this gene in all insect orders indicates that the suppression of proleg development by *abd-A* may have evolved early. However, *Ubx* appears to have appeared at a later time point only in higher insects [38]. Studies have shown that variations in lepidopteran prolegs are common and that they are controlled by *abd-A* [11, 15, 16]. In this study, we also found that *Bmabd-A* is involved in proleg development. It is likely that *abd*-*A* regained the function of specifying lepidopteran proleg development, which is observed in basally branching insects and lost during further evolution.

*Dll* is involved not only in inducing appendage development but also in specifying the distal identities of appendages in arthropods [1, 39–41]. In silkworm, *BmDll* induces proleg development with the absence of *Bmabd-A* in the proleg primordia during early embryogenesis, thus it is suggested that *BmDll* is suppressed by *Bmabd-A* [16, 35]. However, the interaction between Hox and *Dll* in distal appendage structures development is not clear. In *D*. *melanogaster*, *Dll* can induce normal tarsus in thoracic leg development in the absence of Hox function, so it is presumed that Hox genes do not modify the expression of *Dll* that regulates distal appendage structures [42]. But *Proboscipedia* (*Pb*) and *Sex combs reduced* (*Scr*) downregulate *Dll* to give rise to unique distal proboscis structures [43]. Interestingly, the distal structures of prolegs also differ from the thoracic legs, and *Bmabd-A* expression is found in the distal part of proleg primordia in silkworm [15], and overlaps with the expression of *BmDll* [35]. In this study, we also found that the up-regulation of *BmUbx* and down-regulation of *Bmabd-A* expression may lead to the homeotic transformation of A3 to A2 in the *E*^*dl*^ mutant, thus leading to the degenerated abdominal legs phenotype. In other words, the changes in *BmUbx* and *Bmabd-A* expression can alter the expression of *BmDll* in distal prolegs that may be involved in distal proleg development. Spatiotemporal expression patterns of *BmUbx* and *Bmabd-A* in *E*^*dl*^ and Dazao embryos should be studied to confirm this hypothesis.

## Supporting Information

S1 Fig Specificity test and standard curves of *BmUbx* and *Bmabd-A* primers. Standard curves, RT-qPCR product electrophoretogram and melting curve analysis of *Bmabd-A* (A, C, D) and *BmUbx* (B, E, F). We performed a 2-fold serial dilution of a cDNA sample from across the treatment conditions; 2-, 4-, 6- and 16-fold. The concentration at 16-fold dilution with high expression was considered as 16. log_2_. cDNA concentration is represented in the Y axis and Ct is represented in the X axis. All data indicate that *BmUbx* and *Bmabd-A* primers had good efficiency to perform RT-qPCR.
(TIF)

S2 Fig Expression profiles of *BmUbx* and *Bmabd-A* in wildtype and *E*^*dl*^ mutant in stage 20 embryos. RT-qPCR analysis of *Bmabd-A* (A) and *BmUbx* (B). *Bmabd-A* was significantly down-regulated and *BmUbx* was up-regulated in *E*^*dl*^ mutants (Bars indicate mean values ± SD, Student’s *t*-test. *, P<0.05. n = 3). *RPL3* was used as the internal control.
(TIF)

## References

[1] Panganiban G, Sebring A, Nagy L, Carroll S. The development of crustacean limbs and the evolution of arthropods. Science. 1995;270(5240):1363–6. 7481825 10.1126/science.270.5240.1363

[2] Jockusch EL, Williams TA, Nagy LM. The evolution of patterning of serially homologous appendages in insects. Development genes and evolution. 2004;214(7):324–38. 10.1007/s00427-004-0412-6 15170569

[3] Niwa N, Inoue Y, Nozawa A, Saito M, Misumi Y, Ohuchi H, et al Correlation of diversity of leg morphology in *Gryllus bimaculatus* (cricket) with divergence in *dpp* expression pattern during leg development. Development. 2000;127(20):4373–81. 11003837 10.1242/dev.127.20.4373

[4] Snodgrass RE. Principles of Insect Morphology. Ithaca, NY: Cornell University Press; 1935.

[5] Lewis EB. A gene complex controlling segmentation in *Drosophila*. Nature. 1978;276(5688):565–70. 103000 10.1038/276565a0

[6] Lemons D, McGinnis W. Genomic evolution of Hox gene clusters. Science. 2006;313(5795):1918–22. 10.1126/science.1132040 17008523

[7] Vachon G, Cohen B, Pfeifle C, McGuffin ME, Botas J, Cohen SM. Homeotic genes of the Bithorax complex repress limb development in the abdomen of the *Drosophila* embryo through the target gene *Distal-less*. Cell. 1992;71(3):437–50. 1358457 10.1016/0092-8674(92)90513-c

[8] Castelligair J, Akam M. How the Hox Gene *Ultrabithorax* Specifies 2 Different Segments: the Significance of Spatial and Temporal Regulation within Metameres. Development. 1995;121(9):2973–82. 7555723 10.1242/dev.121.9.2973

[9] Estrada B, Sanchez-Herrero E. The Hox gene *Abdominal-B* antagonizes appendage development in the genital disc of *Drosophila*. Development. 2001;128(3):331–9. 11152632 10.1242/dev.128.3.331

[10] Lewis DL, DeCamillis M, Bennett RL. Distinct roles of the homeotic genes *Ubx* and *abd-A* in beetle embryonic abdominal appendage development. Proceedings of the National Academy of Sciences of the United States of America. 2000;97(9):4504–9. 10781052 10.1073/pnas.97.9.4504 PMC18264

[11] Zheng Z, Khoo A, Fambrough D Jr., Garza L, Booker R. Homeotic gene expression in the wild-type and a homeotic mutant of the moth *Manduca sexta*. Development genes and evolution. 1999;209(8):460–72. 10415323 10.1007/s004270050279

[12] Masumoto M, Yaginuma T, Niimi T. Functional analysis of *Ultrabithorax* in the silkworm, *Bombyx* mori, using RNAi. Development genes and evolution. 2009;219(9–10):437–44. 10.1007/s00427-009-0305-9 19908062

[13] Pan MH, Wang XY, Chai CL, Zhang CD, Lu C, Xiang ZH. Identification and function of *Abdominal-A* in the silkworm, *Bombyx mori*. Insect molecular biology. 2009;18(2):155–60. 10.1111/j.1365-2583.2009.00862.x 19320756

[14] Xiang H, Li MW, Guo JH, Jiang JH, Huang YP. Influence of RNAi knockdown for E-complex genes on the silkworm proleg development. Archives of insect biochemistry and physiology. 2011;76(1):1–11. 10.1002/arch.20393 21125568

[15] Chen P, Tong XL, Li DD, Liang PF, Fu MY, Li CF, et al Fine mapping of a supernumerary proleg mutant (*E*^*Cs*^ *-l*) and comparative expression analysis of the *abdominal-A* gene in silkworm, *Bombyx mori*. Insect molecular biology. 2013;22(5):497–504. 10.1111/imb.12039 23803144

[16] Tomita S, Kikuchi A. *Abd-B* suppresses lepidopteran proleg development in posterior abdomen. Developmental biology. 2009;328(2):403–9. 10.1016/j.ydbio.2009.01.040 19389347

[17] Ueno K, Hui CC, Fukuta M, Suzuki Y. Molecular analysis of the deletion mutants in the E homeotic complex of the silkworm *Bombyx mori*. Development. 1992;114(3):555–63. 1352236 10.1242/dev.114.3.555

[18] Maeda RK, Karch F. The ABC of the BX-C: the bithorax complex explained. Development. 2006;133(8):1413–22. 10.1242/dev.02323 16556913

[19] Gummalla M, Maeda RK, Castro Alvarez JJ, Gyurkovics H, Singari S, Edwards KA, et al *abd-A* regulation by the *iab-8* noncoding RNA. PLoS genetics. 2012;8(5):e1002720 10.1371/journal.pgen.1002720 22654672 PMC3359974

[20] Bender W. MicroRNAs in the *Drosophila* bithorax complex. Genes & development. 2008;22(1):14–9. 18172161 10.1101/gad.1614208 PMC2151010

[21] Maeda RK, Karch F. The bithorax complex of *Drosophila* an exceptional Hox cluster. Current topics in developmental biology. 2009;88:1–33. 10.1016/S0070-2153(09)88001-0 19651300

[22] Zhou J, Levine M. A novel *cis*-regulatory element, the PTS, mediates an anti-insulator activity in the *Drosophila* embryo. Cell. 1999;99(6):567–75. 10612393 10.1016/s0092-8674(00)81546-9

[23] Xiang H, Li M, Yang F, Guo Q, Zhan S, Lin H, et al Fine mapping of *E*^*kp*^*-*1, a locus associated with silkworm (*Bombyx mori*) proleg development. Heredity (Edinb). 2008;100(5):533–40. 18364737 10.1038/hdy.2008.10

[24] Yasukochi Y, Ashakumary LA, Wu C, Yoshido A, Nohata J, Mita K, et al Organization of the Hox gene cluster of the silkworm, *Bombyx mori*: a split of the Hox cluster in a non-*Drosophila* insect. Development genes and evolution. 2004;214(12):606–14. 10.1007/s00427-004-0441-1 15490231

[25] Xiang ZH. Biology of Sericulture. Beijing (in Chinese): China Forestry Publishing House; 2005.

[26] Takami T, Kitazawa T. Normal stages of the embryonic development in the silkworm, *Bombyx mori*. Tech Bull Sericult Exp Sta 1960;75:1–31.

[27] Altschul SF, Madden TL, Schaffer AA, Zhang J, Zhang Z, Miller W, et al Gapped BLAST and PSI-BLAST: a new generation of protein database search programs. Nucleic acids research. 1997;25(17):3389–402. 9254694 10.1093/nar/25.17.3389 PMC146917

[28] Tong X, Hrycaj S, Podlaha O, Popadic A, Monteiro A. Over-expression of *Ultrabithorax* alters embryonic body plan and wing patterns in the butterfly *Bicyclus anynana*. Developmental biology. 2014;394(2):357–66. 10.1016/j.ydbio.2014.08.020 25169193

[29] Yu X, Zhou Q, Li SC, Luo Q, Cai Y, Lin WC, et al The silkworm (*Bombyx mori*) microRNAs and their expressions in multiple developmental stages. PloS one. 2008;3(8):e2997 10.1371/journal.pone.0002997 18714353 PMC2500172

[30] Huang Y, Zou Q, Tang SM, Wang LG, Shen XJ. Computational identification and characteristics of novel microRNAs from the silkworm (*Bombyx mori* L.). Molecular biology reports. 2010;37(7):3171–6. 10.1007/s11033-009-9897-4 19823945

[31] Liu S, Li D, Li Q, Zhao P, Xiang Z, Xia Q. MicroRNAs of *Bombyx mori* identified by Solexa sequencing. BMC Genomics. 2010;11:148 10.1186/1471-2164-11-148 20199675 PMC2838851

[32] Cohen SM, Bronner G, Kuttner F, Jurgens G, Jackle H. *Distal-less* encodes a homoeodomain protein required for limb development in *Drosophila*. Nature. 1989;338(6214):432–4. 10.1038/338432a0 2564639

[33] Beermann A, Jay DG, Beeman RW, Hulskamp M, Tautz D, Jurgens G. The *Short antennae* gene of Tribolium is required for limb development and encodes the orthologue of the Drosophila *Distal-less* protein. Development. 2001;128(2):287–97. 11124123 10.1242/dev.128.2.287

[34] Schoppmeier M, Damen WG. Double-stranded RNA interference in the spider *Cupiennius salei*: the role of *Distal-less* is evolutionarily conserved in arthropod appendage formation. Development genes and evolution. 2001;211(2):76–82. 11455417 10.1007/s004270000121

[35] Singh A, Kango-Singh M, Parthasarathy R, Gopinathan KP. Larval legs of mulberry silkworm *Bombyx mori* are prototypes for the adult legs. Genesis. 2007;45(4):169–76. 10.1002/dvg.20280 17417803

[36] Warren RW, Nagy L, Selegue J, Gates J, Carroll S. Evolution of homeotic gene regulation and function in flies and butterflies. Nature. 1994;372(6505):458–61. 10.1038/372458a0 7840822

[37] Konopova B, Akam M. The Hox genes *Ultrabithorax* and *abdominal-A* specify three different types of abdominalappendage in the springtail *Orchesella cincta* (Collembola). EvoDevo. 2014;5(1):2 10.1186/2041-9139-5-2 24398075 PMC3910676

[38] Palopoli MF, Patel NH. Evolution of the interaction between Hox genes and a downstream target. Current biology: CB. 1998;8(10):587–90. 9601643 10.1016/s0960-9822(98)70228-3

[39] Cohen SM. Specification of limb development in the *Drosophila* embryo by positional cues from segmentation genes. Nature. 1990;343(6254):173–7. 10.1038/343173a0 2296309

[40] Panganiban G, Nagy L, Carroll SB. The role of the *Distal-less* gene in the development and evolution of insect limbs. Current biology: CB. 1994;4(8):671–5. 7953552 10.1016/s0960-9822(00)00151-2

[41] Cohen SM, Jurgens G. Proximal-distal pattern formation in *Drosophila*: cell autonomous requirement for *Distal-less* gene activity in limb development. The EMBO journal. 1989;8(7):2045–55. 16453891 10.1002/j.1460-2075.1989.tb03613.x PMC401088

[42] Casares F, Mann RS. The ground state of the ventral appendage in *Drosophila*. Science. 2001;293(5534):1477–80. 10.1126/science.1062542 11520984

[43] Abzhanov A, Holtzman S, Kaufman TC. The *Drosophila* proboscis is specified by two Hox genes, *proboscipedia* and *Sex combs reduced*, via repression of leg and antennal appendage genes. Development. 2001;128(14):2803–14. 11526085 10.1242/dev.128.14.2803

