# Supplementary figures and images for: Fine Mapping of a Degenerated Abdominal Legs Mutant (Edl) in Silkworm, Bombyx mori
Source: PLoS One. 2017 Jan 12;12(1):e0169224. doi: 10.1371/journal.pone.0169224 (PMC5231277; doi:10.1371/journal.pone.0169224)

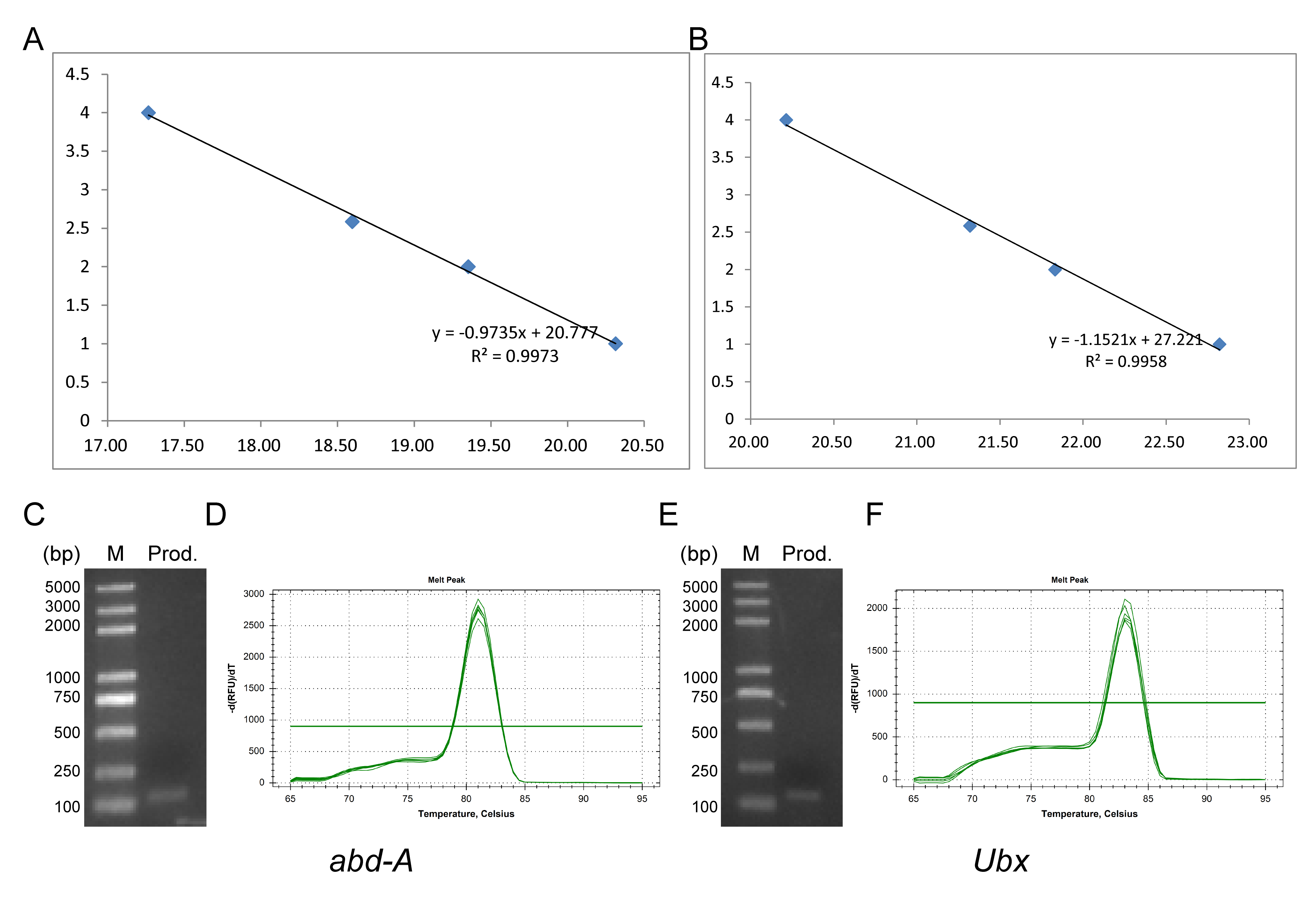

Supplement: S1 Fig — Standard curves, RT-qPCR product electrophoretogram and melting curve analysis of Bmabd-A (A, C, D) and BmUbx (B, E, F). We performed a 2-fold serial dilution of a cDNA sample from across the treatment conditions; 2-, 4-, 6- and 16-fold. The concentration at 16-fold dilution with high expression was considered as 16. log2. cDNA concentration is represented in the Y axis and Ct is represented in the X axis. All data indicate that BmUbx and Bmabd-A primers had good efficiency to perform RT-qPCR. (TIF) [file pone.0169224.s001.tif]

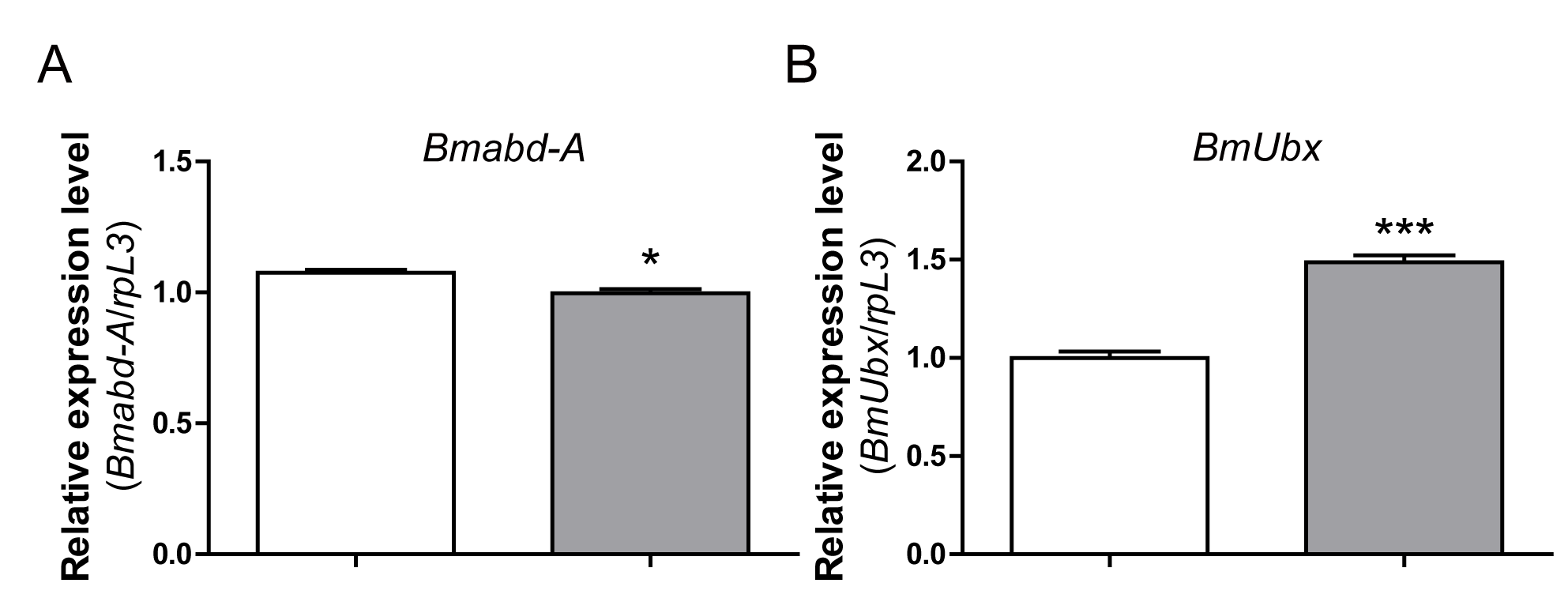

Supplement: S2 Fig — RT-qPCR analysis of Bmabd-A (A) and BmUbx (B). Bmabd-A was significantly down-regulated and BmUbx was up-regulated in Edl mutants (Bars indicate mean values ± SD, Student’s t-test. *, P<0.05. n = 3). RPL3 was used as the internal control. (TIF) [file pone.0169224.s002.tif]
